# Neurog1-Derived Peptides RMNE1 and DualPep-Shine Penetrate the Skin and Inhibit Melanin Synthesis by Regulating MITF Transcription

**DOI:** 10.3390/ijms24076158

**Published:** 2023-03-24

**Authors:** Ee Chan Song, Chanho Park, Yungyeong Shin, Wan Ki Kim, Sang Bum Kim, Seongmin Cho

**Affiliations:** 1 Remedi Co., Ltd., Research Center, Incheon 21990, Republic of Korea; chio5252@re-medi.co.kr (E.C.S.); chanhp@re-medi.co.kr (C.P.); ygshin@re-medi.co.kr (Y.S.); dhksrl9999@re-medi.co.kr (W.K.K.); 2 College of Pharmacy, Sahmyook University, Seoul 01795, Republic of Korea; sbk@syu.ac.kr

**Keywords:** cell-penetrating peptide, Neurog1, melanin, microphthalmia-associated transcription factor, Neoderm, skin penetration, DualPep-Shine

## Abstract

Anti-pigmentation peptides have been developed as alternative skin-lightening agents to replace conventional chemicals that have adverse effects on the skin. However, the maximum size of these peptides is often limited by their low skin and cell penetration. To address this issue, we used our intra-dermal delivery technology (IDDT) platform to identify peptides with hypo-pigmenting and high cell-penetrating activity. Using our cell-penetrating peptides (CPPs) from the IDDT platform, we identified RMNE1 and its derivative RMNE3, “DualPep-Shine”, which showed levels of α-Melanocyte stimulating hormone (α-MSH)-induced melanin inhibition comparable to the conventional tyrosinase inhibitor, Kojic acid. In addition, DualPep-Shine was delivered into the nucleus and regulated the gene expression levels of melanogenic enzymes by inhibiting the promoter activity of microphthalmia-associated transcription factor-M (MITF-M). Using a 3D human skin model, we found that DualPep-Shine penetrated the lower region of the epidermis and reduced the melanin content in a dose-dependent manner. Furthermore, DualPep-Shine showed high safety with little immunogenicity, indicating its potential as a novel cosmeceutical ingredient and anti-pigmentation therapeutic agent.

## 1. Introduction

Melanin generation from melanocytes is essential for protecting the skin from ultraviolet (UV) light and scavenging chemicals [1,2]. However, melanin accumulation often causes skin disorders such as melisma, freckles, and age spots [3,4]. Therefore, the discovery and development of active hypopigmenting agents have been extensively investigated. In particular, chemicals such as Arbutin and Kojic acid, which target tyrosinase, have been studied and tested in cosmetics to regulate melanin synthesis [5,6,7]. However, their utilization on the skin is limited because of their poor efficacy, low skin penetration, and adverse effects in vivo. In this regard, finding new whitening agents with potent efficacy and high safety is required for cosmetics and pigment diseases [8,9].

As alternative whitening agents, peptides have been tested for their efficacy in regulating skin pigmentation [10,11]. These peptides can suppress melanin synthesis by targeting the receptors, signaling molecules, and enzymes involved in melanin and melanosome synthesis [12,13]. These anti-pigmentation peptides usually contain 2–20 amino acids (aa) and often show better efficacy than chemicals or natural compounds with a larger surface area for binding specific targets [14,15,16]. However, it is already known that their molecular size is very limited due to their lower permeability [17].

Cell-penetrating peptides (CPPs), usually comprising 5–25 aa, are widely known to efficiently cross membranes, despite their high molecular weight. Furthermore, these peptides usually carry cationic charges and are attached to various cargo molecules for cellular and skin delivery. Therefore, many CPPs have been tested for their potential to increase the delivery of low-absorbing ingredients [18,19,20,21]. However, despite extensive research on its role in transmembrane delivery, the intracellular biological activities of CPPs are yet to be fully investigated [22]. Since these peptides have relatively large surface areas and carry cationic charges, we predicted that their biological activities manifest once they penetrate cells and that some CPPs may interfere with melanin synthesis [23,24,25,26]. Therefore, in this study, we tested a range of CPPs for possible anti-melanogenic function to determine their potential as whitening agents.

## 2. Results

### 2.1. Using CPPs for Melanin Inhibition Screening in B16F10 Cells

**Figure 1:**
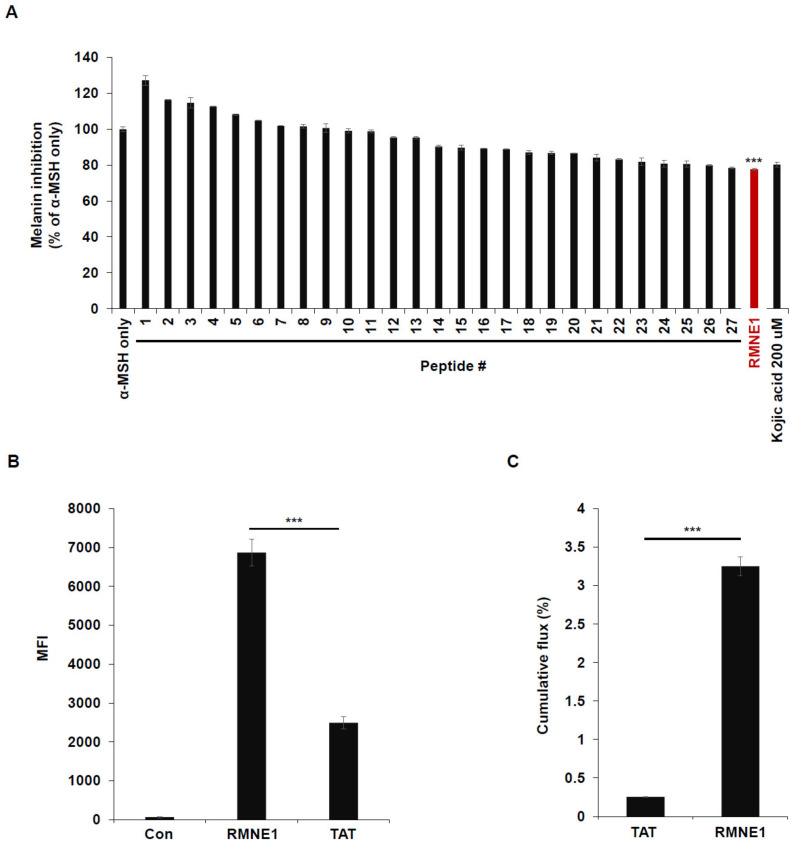
Using CPPs for melanin inhibition screening in B16F10 cells. (**A**) Melanin inhibition was tested after incubating B16F10 with α-MSH (50 nM) and CPPs (20 μM) for 72 h. As a positive control, 200 μM of Kojic acid was used. Melanin contents were measured at optical density (OD) 405 nm. (**B**) B16F10 cells were treated with 2.5 μM of FITC-conjugated RMNE1 for 2 h. The mean fluorescence intensity (MFI) of the cells was determined via flow cytometry analysis. FITC-conjugated TAT was used to compare the uptake efficiency. (**C**) Franz diffusion cells and artificial membranes were used to test the skin penetration effect. Totals of 500 μL of 1 mM FITC-conjugated RMNE1 and TAT were placed in the donor compartment of Franz cells for 24 h. Penetrated peptides were collected from the receiver compartment, and the concentrations of each sample were calculated using a fluorescence reader. Results are presented as mean ± SD (standard deviation), and statistical significance was analyzed with Student’s *t*-test (*** *p* < 0.001).

Previously, we developed an intra-dermal delivery technology (IDDT) platform that can deliver cargo to cells more effectively than the TAT (transactivator of transcription) peptide can. To explore the effects of these CPPs on melanin synthesis, we treated B16F10 cells with α-Melanocyte stimulating hormone (α-MSH) and 28 different CPPs from our IDDT platform. Kojic acid has been previously reported to reduce melanin synthesis by acting as a potent tyrosinase inhibitor; we therefore used Kojic acid as a control to compare the efficacy of melanin inhibition by the CPPs [27,28]. As a result, we found that CPP derived from Neurog1 (RMNE1) showed melanin inhibition comparable to that by Kojic acid and much higher than that by other CPPs tested (Figure 1A). Next, to confirm that RMNE1 was able to penetrate cells in vitro, we treated B16F10 cells with the fluorescein isothiocyanate (FITC)-conjugated RMNE1 peptide and checked the fluorescence levels of penetrated peptides after several washes and trypsin treatment to remove membrane-bound peptides. As a result, we found, using flow cytometry analysis, that RMNE1 was delivered at a much higher level than the TAT peptide (Figure 1B). We also tested skin penetration using artificial membranes and Franz cells, a well-studied model for assessing skin permeation [29,30]. After treating the donor cells with FITC-conjugated RMNE1 or the TAT peptide, samples were collected from the receiver cells 24 h later. The results showed that RMNE1 was found in receiver cells at much higher levels than the TAT peptide (Figure 1C). Through anti-pigmentation screening using our CPPs, we found that RMNE1 inhibited melanin synthesis comparably to Kojic acid and penetrated cells and skin better than the TAT peptide.

### 2.2. RMNE1 and Its Derivatives Inhibit Melanin Synthesis by Suppressing Melanogenesis-Related Enzymes

**Figure 2:**
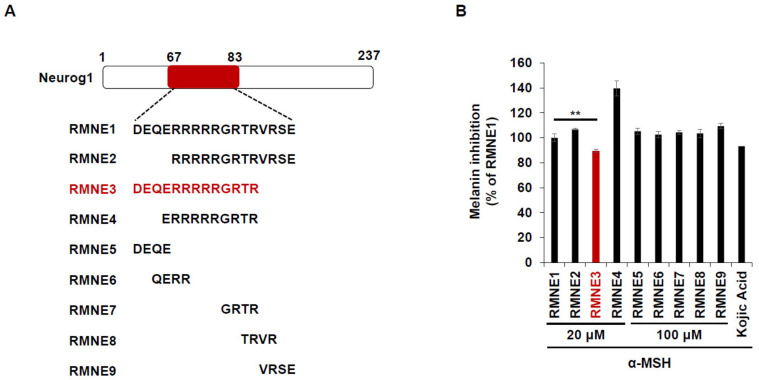

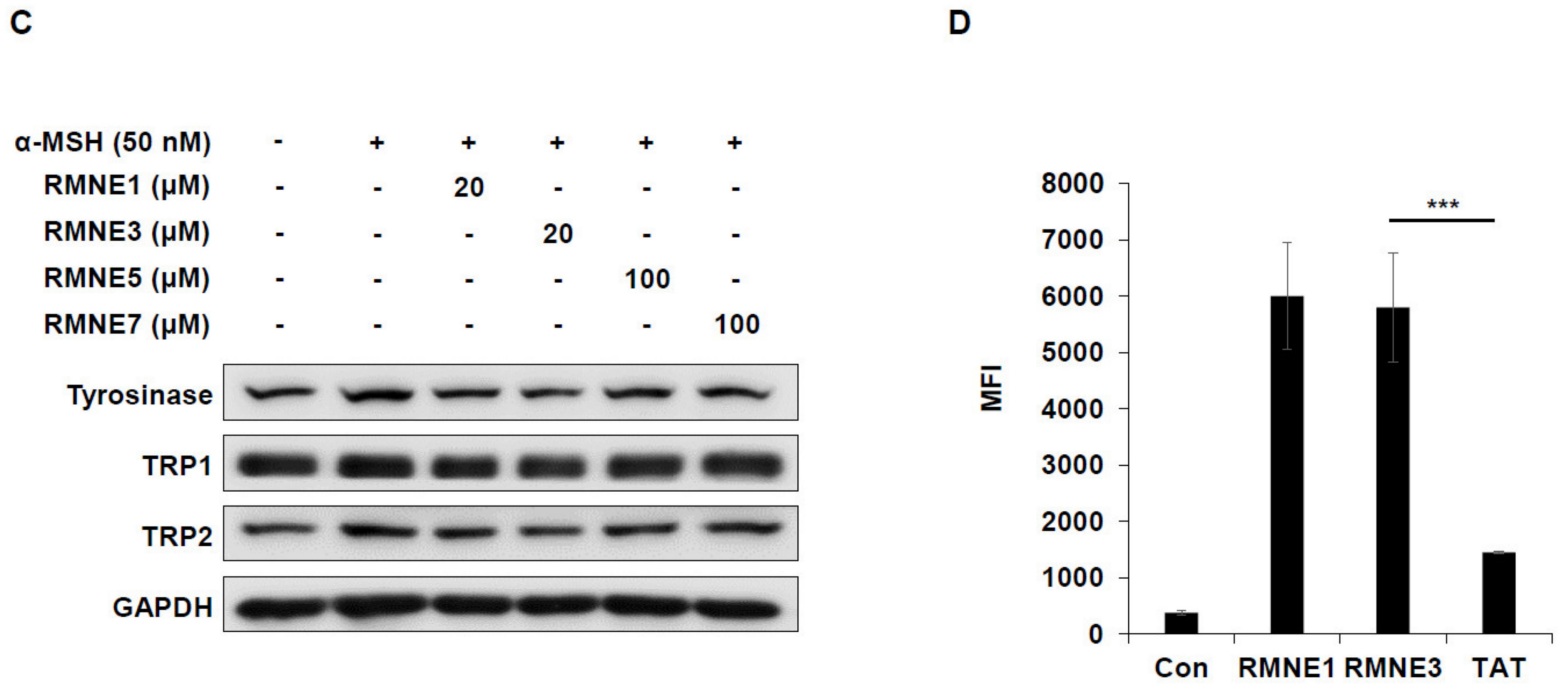
RMNE1 and its derivatives inhibit melanin synthesis by suppressing melanogenesis-related enzymes. (**A**) Schematic demonstration of fragmented RMNE1 peptides. (**B**) Melanin inhibition was measured after treatment with α-MSH (50 nM) and RMNE1 derivatives on B16F10 cells. As positive control, 200 μM of Kojic acid was used. (**C**) B16F10 cells were incubated with RMNE1 derivatives with indicated concentrations and α-MSH for 24 h. Whole-cell lysates were subjected to Western blotting, and indicative antibodies were used to detect each protein. GAPDH (glyceraldehyde 3-phosphate dehydrogenase) was used as the loading control. (**D**) B16F10 cells were treated with 2.5 μM of FITC-conjugated RMNE1 and RMNE3 for 2 h. The mean fluorescence intensity (MFI) of the cells was determined via flow cytometry analysis. The results are presented as mean ± SD, and statistical significance was analyzed with Student’s *t*-test (** *p* < 0.01, *** *p* < 0.001).

Next, we synthesized derivatives of RMNE1 by deleting N- and C-terminal regions and creating four amino acid fragments to enhance the melanin inhibitory effects. Among the various RMNE1 derivatives, RMNE3, a derivative with a C-terminal deletion (aa “VRSE” deletion form), showed the most potent melanin inhibition (Figure 2A,B). Moreover, we treated the cells with the most potent RMNE1 derivatives (RMNE3, RMNE5, and RMNE7) to determine the molecular changes in melanogenesis-related proteins [31]. Like the melanin inhibition results, the expression levels of Tyrosinase, TRP1 (tyrosinase related protein 1), and TRP2 were suppressed by RMNE1 derivatives (Figure 2C). In particular, we found that RMNE3 reduced the expression levels of melanogenesis-related proteins the most. Then, to determine whether RMNE3 showed increased melanin inhibition because of enhanced cell penetration, we compared the cell penetration abilities of RMNE1 and RMNE3 using flow cytometry analysis. We found that RMNE1 and RMNE3 showed similar penetration abilities (Figure 2D), indicating that the efficacy of RMNE3 may be due to subtle changes in peptide structure rather than changes in cell penetration ability. Overall, we found that RMNE1 and its derivative RMNE3 showed potent melanin inhibition while possessing cell-penetrating ability. We named the final functional RMNE3 derivative “DualPep-Shine”.

### 2.3. DualPep-Shine Regulates the Expression of Melanogenic Genes and Inhibits the Promoter Activity of MITF-M

**Figure 3:**
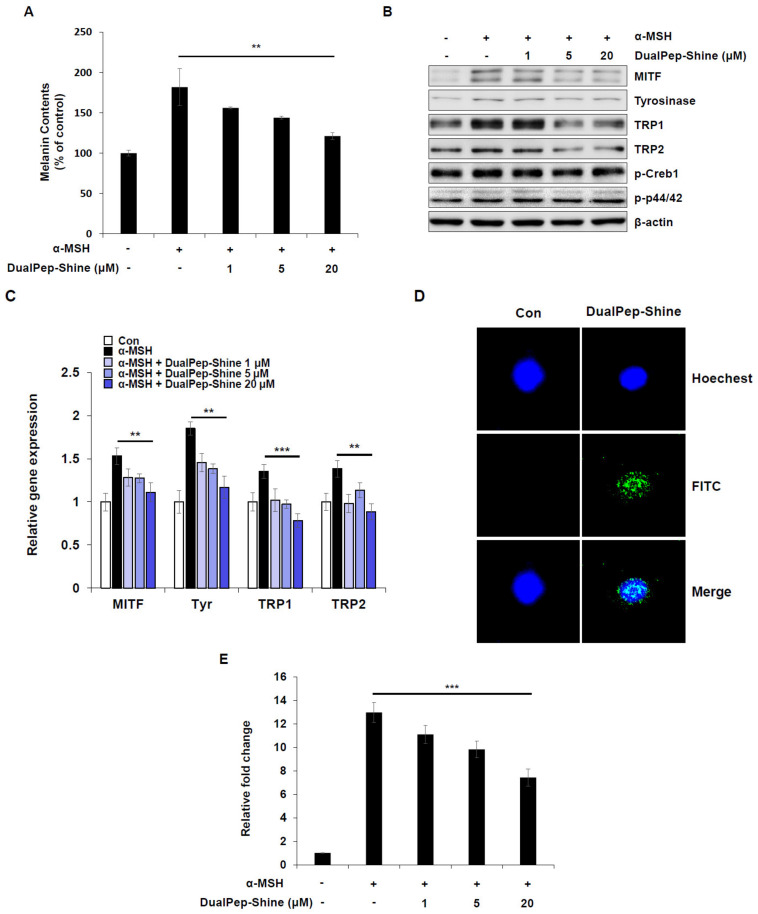
DualPep-Shine regulates the expression of melanogenic genes and inhibits the promoter activity of MITF-M. (**A**) B16F10 cells were treated with various concentrations of DualPep-Shine for 72 h. Melanin contents were determined at OD 405 nm. (**B**) DualPep-Shine was treated with α-MSH for 24 h, and whole-cell lysates were subjected to Western blotting. Indicated antibodies were used to analyze melanogenic proteins. (**C**) mRNA levels of MITF, Tyr, TRP1, and TRP2 were analyzed using quantitative polymerase chain reaction (qPCR) after treatment with DualPep-Shine for 12 h. The gene expression was normalized with β-actin. (**D**) FITC-conjugated DualPep-Shine was treated for 2 h on B16F10 cells, and its localization was visualized using confocal microscopy. Hoechst 33342 was used for nucleus staining. Bar = 10 μm. (**E**) Luciferase reporter assay was performed to check the promoter activity of MITF-M. Cells were transfected with MITF-M promoter construct and treated with DualPep-Shine for 8 h. The results are presented as mean ± SD, and statistical significance was analyzed with Student’s *t*-test (** *p* < 0.01, *** *p* < 0.001).

To confirm and understand the mechanism of melanin inhibition by DualPep-Shine, we treated B16F10 cells with α-MSH and different concentrations of DualPep-Shine. As a result, we found that DualPep-Shine treatment decreased α-MSH-mediated melanin synthesis in a dose-dependent manner, reducing it by 31%, 46%, and 74% at 1, 5, and 20 uM, respectively (Figure 3A). Additionally, we measured the relative expression levels of melanogenesis-related proteins after stimulating B16F10 cells with α-MSH and different concentrations of DualPep-Shine and found that the expression levels of MITF, Tyrosinase, TRP1, and TRP were decreased by DualPep-Shine in a dose-dependent manner (Figure 3B). Next, we investigated the expression levels of melanogenic genes to explore the underlying mechanism of DualPep-Shine. We found that the expression levels of melanogenic genes, including MITF, Tyrosinase, TRP1, and TRP2, decreased in a dose-dependent manner after DualPep-Shine treatment (Figure 3C). As DualPep-Shine altered the gene expression levels, we examined signaling pathways and DualPep-Shine localization. DualPep-Shine treatment did not change the phosphorylation levels of p44/42 and Cathelicidin Antimicrobial Peptide responsive element binding protein 1 (CREB1), indicating that it does not interfere with the α-MSH-induced signaling pathway (Figure 3B) [32]. Moreover, we found that DualPep-Shine was mainly localized in the nucleus when visualized using confocal microscopy (Figure 3D). As DualPep-Shine decreases the expression level of MITF mRNA and is localized in the nucleus, we next examined whether DualPep-shine regulates MITF-M promoter activity. We found that α-MSH-mediated MITF-M promoter activity was suppressed by DualPep-Shine in a dose-dependent manner (Figure 3E). These findings indicate that DualPep-Shine decreases the melanin levels in cells by entering the nucleus and regulating the expression of melanogenesis-related genes by inhibiting MITF-M promoter activity.

### 2.4. Inhibitory Effect of DualPep-Shine on Melanin Synthesis in Neoderm-ME Human Skin

**Figure 4:**
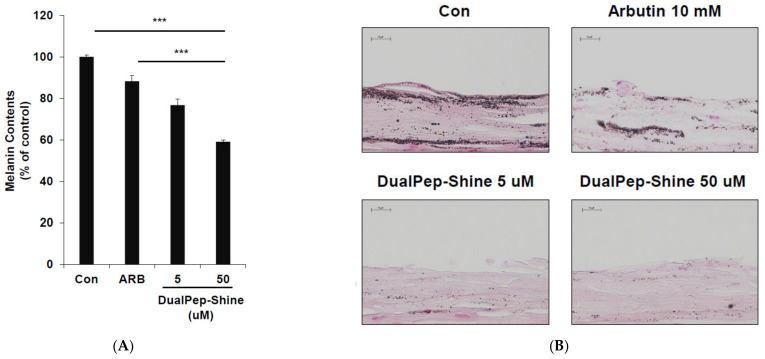

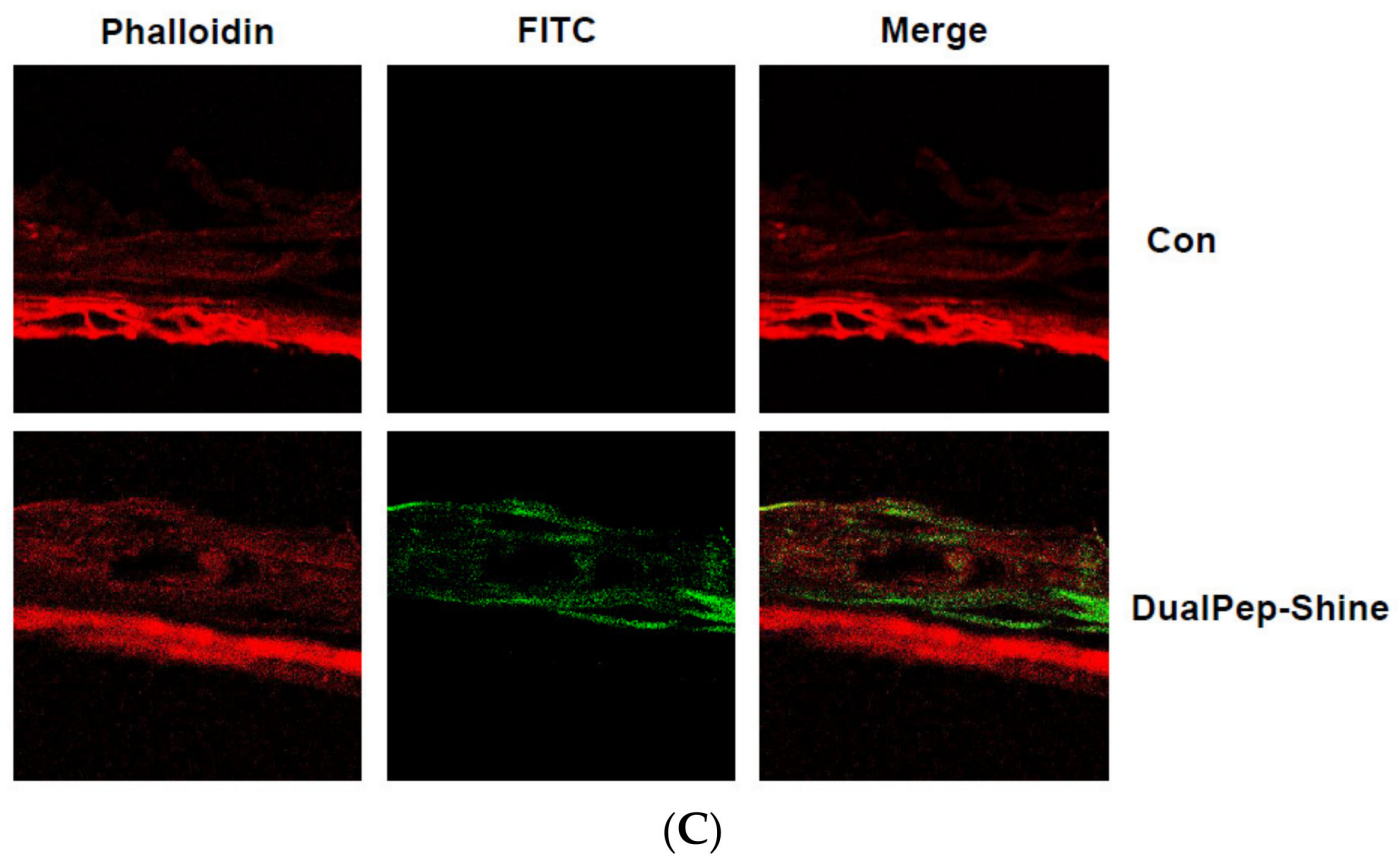
Inhibitory effect of DualPep-Shine on melanin synthesis in Neoderm-ME human skin model. Neoderm-ME was treated with indicated concentrations of DualPep-Shine. Arbutin was used as a positive control. (**A**) Melanin contents were measured at OD 405 nm. (**B**) Melanin contents were visualized via Fontana–Masson staining. Representative images are shown. Bar = 40 μm. (**C**) Representative confocal microscopy images of the Neoderm-ME after the treatment with FITC-conjugated DualPep-Shine for 24 h. Alexa Fluro 555 phalloidin was used to visualize the epidermis. Bar = 40 μm. The results are presented as mean ± SD, and statistical significance was analyzed with Student’s *t*-test (*** *p* < 0.001).

Next, we assessed the melanin-inhibiting effect of DualPep-Shine in a 3D human skin model [33,34,35]. As 3D human skin (Neoderm-ME) contains human keratinocytes and melanocytes and shows melanogenesis over time, we measured the melanin content in Neoderm-ME after incubation with different concentrations of DualPep-Shine or Arbutin (a known regulator of melanin synthesis) for 6 days. We found that DualPep-Shine reduced the melanin content in a dose-dependent manner and attenuated melanin synthesis to a greater extent than Arbutin, suggesting its superiority in inhibiting melanin synthesis (Figure 4A). Additionally, the tissue section images obtained via Fontana–Masson staining show that the melanin deposition in tissue was profoundly decreased by DualPep-Shine treatment (Figure 4B). Moreover, we used FITC-conjugated DualPep-Shine to validate the skin-penetrating ability of DualPep-Shine. After treating the FITC-conjugated DualPep-Shine for 24 h, confocal images were analyzed to check the penetration of DualPep-Shine. Consistent with the Franz cell assay using artificial skin, FITC-DualPep-Shine penetrated the skin and was detected in the lower part of the epidermis (Figure 4C). Based on these results, we concluded that DualPep-Shine could penetrate the epidermis and inhibit melanin synthesis in a 3D human skin model, suggesting DualPep-Shine would reduce melanin production on human skin.

### 2.5. DualPep-Shine Shows High Stability with Little Toxicity

**Figure 5:**
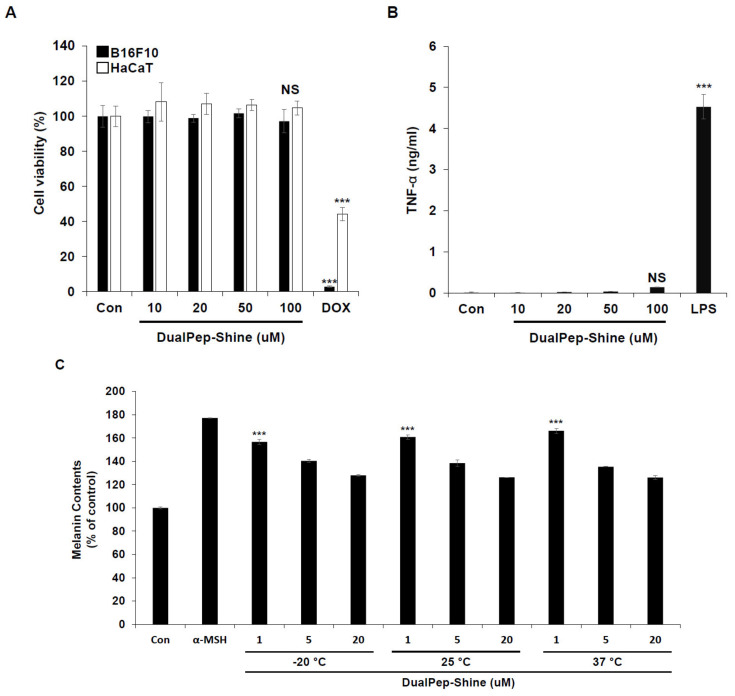
DualPep-Shine shows high stability with little toxicity. (**A**) B16F10 and HaCaT cells were treated with the indicated concentration of DualPep-Shine for 72 h, and relative cell viability was measured using Cell Counting Kit 8 (CCK8) assay. Doxorubicin was used for positive control. (**B**) Phorbol 12-myristate 13-acetate (PMA)-differentiated THP-1 cells were treated with different concentrations of DualPep-Shine for 24 h. TNF-α from supernatants was measured using the enzyme-linked immunosorbent assay (ELISA). (**C**) DualPep-Shine was incubated with indicated temperature conditions for 45 days. B16F10 cells were treated with different concentrations for 72 h, and melanin contents were measured at OD 405 nm. The results are presented as mean ± SD, and statistical significance was analyzed with Student’s *t*-test (NS: not significant, *** *p* < 0.001).

As keratinocytes and melanocytes are the most abundant cell types in the outer part of the skin, we investigated the toxicity of DualPep-Shine by incubating HaCaT and B16F10 cells with different concentrations of the peptide. Cell viability was not affected, even at the highest concentration of DualPep-Shine, indicating that the anti-pigmentation effect of the peptide was unrelated to cell viability (Figure 5A). Additionally, we assessed the immunogenicity of DualPep-Shine and found that it did not induce tumor necrosis factor α (TNF-α) secretion even during 100 μM DualPep-Shine treatment (Figure 5B). Furthermore, to test the stability of DualPep-Shine, we pre-incubated the peptide at various temperatures (−20 °C, 25 °C, and 37 °C) for 45 days. After the pre-incubation, the anti-melanin synthesis activity of DualPep-Shine was determined by measuring the melanin contents of B16F10 cells. We found that the anti-pigmentation activity of DualPep-Shine was maintained after incubation at different temperatures for 45 days (Figure 5C). These data suggest that DualPep-Shine is non-toxic and stable, supporting its use as a cosmeceutical ingredient.

## 3. Discussion

In this study, we identified RMNE1 and DualPep-Shine, peptides with anti-pigmenting activity derived from human Neurog1. Neurog1 belongs to a family of basic helix–loop–helix transcription factors that regulate the differentiation of ganglion neurons during development [36,37]. Considering that Neurog1 is mainly expressed during neuronal differentiation and is seen at very low levels in other tissues, it is unclear whether Neurog1 has an endogenous function in melanocytes [38,39]. The anti-pigmenting activity and nucleus localization of RMNE1 and DualPep-Shine may provide a clue for Neurog1 biology in melanocytes, but the precise mechanism for the anti-pigmenting activity and biological function should be further investigated.

Several biologically active peptides and proteins have been developed for topical use; however, the use of peptides on the skin is limited because their large molecular weights are often not conducive to skin permeation. Many CPPs have been studied for their ability to facilitate the uptake of active ingredients, including peptides and proteins. For instance, CPPs from exogenous sources, such as TAT (GRKKRRQRRRPQ) and penetratin (RQIKIWFQNRRMKWKK), are widely used to deliver active proteins into various cell types [40,41]. In addition, arginine-rich peptides, especially the R7 peptide (RRRRRRR), showed skin-penetrating abilities when connected to an anti-inflammatory peptide, cyclosporine A [42]. These observations suggest that CPPs can penetrate skin and cells, even with their large molecular size [43,44]. Thus, the high penetration ability of RMNE1 and DualPep-Shine supports their potential for topical use, especially in the cosmetics field.

Through flow cytometry and immunofluorescence analyses, we found that DualPep-Shine was efficiently delivered into cells and was detected in both the cytosol and the nucleus. To examine the mechanism of pigmentation regulation, we quantified melanogenesis-related enzymes and signaling molecules using qPCR and Western blotting. We found that the gene expression levels of enzymes related to melanin synthesis were all altered, while levels of signaling molecules driven by α-MSH were not altered by DualPep-Shine treatment. These results suggest that DualPep-Shine does not inhibit melanogenesis by interfering with signaling molecules in the cytosol but by regulating melanogenesis genes in the nucleus. In particular, DualPep-Shine inhibited the promoter activity of MITF-M, which regulates the expression of melanogenesis-related enzymes. These data suggest that DualPep-Shine controls MITF transcription once localized in the nucleus. It is possible that DualPep-Shine disrupts MITF transcription by binding to the promoter region of MITF with its basic residues or interacting with co-activators and transcription factors related to the MITF-M promoter [45,46,47]; however, further studies are required to determine the precise mechanism of action.

The use of peptides as functional ingredients in the cosmetic industry has rapidly increased. Peptides have demonstrated low toxicity and irritation on human skin tissue while maintaining great stability. As a result, various bio-functional peptides have been clinically tested for their cosmeceutical activities. However, many compounds and peptides that may exhibit cosmetic activities are constrained by their inability to penetrate cellular membranes. Considering that DualPep-Shine effectively penetrates cell membranes and demonstrates anti-pigmenting activity in a 3D skin model that mimics human tissue, it may be effective in a clinical trial for skin depigmentation. Prior to clinical application, additional studies will be conducted to determine the formulation of DualPep-Shine and its synergistic effects with other ingredients.

Here, we used our IDDT platform, which consists of potential and validated CPPs, to identify active peptides that can reduce melanin biogenesis. Throughout the melanin inhibition assay, we found that RMNE1 and its derivative RMNE3, “DualPep-Shine”, showed potent anti-pigmenting activity compared with conventional anti-pigmenting agents such as Arbutin and Kojic acid. Additionally, its potency and safety were validated using flow cytometry, Franz cells, and 3D skin models. Based on this report on CPPs as a functional ingredient, we suggest its use as a hypo-pigmenting agent in cosmetics and pharmaceuticals.

## 4. Materials and Methods

### 4.1. Cell Viability Assay

Cell viability was measured using a CCK-8 assay (Dojindo, Tokyo, Japan). HaCaT and B16F10 cells were cultured in a 96-well plate at a 6 × 10^3^ cells/well and incubated in a complete medium at 37 °C with 5% CO_2_ overnight. Each cell type was treated with different concentrations of peptides for 24 h. After removing the medium, CCK-8 solution was added in a dark room at 37 °C. Absorbance was measured at 450 nm using a microplate reader.

### 4.2. Peptide Synthesis

All the peptides, including TAT, CPPs, RMNE1, and RMNE1 derivatives, were synthesized following standard Fmoc-SPPS (solid phase peptide synthesis) protocols using chloride resin and amino acids. Peptides were cleaved from the resin, and side-chain protecting groups were removed from amino acids using trifluoroacetic acid (TFA, Sigma-Aldrich, Darmstadt, Germany), water, and triisopropylsilane (Sigma-Aldrich, Darmstadt, Germany). Peptides were purified by the reverse phase using a C18 column and eluted with a water–acetonitrile linear gradient containing 0.1% TFA. The molecular weights of the purified peptides were confirmed using LC/MS.

### 4.3. Western Blot Analysis

B16F10 cells (3 × 10^5^) were seeded in a 6-well plate and incubated for 16 h. The cells were treated with each peptide and cultured at 37 °C in 5% CO_2_. The cells were scraped in a RIPA buffer (Biosesang, Seongnam, Republic of Korea) with a protease inhibitor cocktail (Merck, Darmstadt, Germany) and incubated on ice for 30 min. After the cell lysate mixture was centrifuged at 13,000 rpm for 15 min, the supernatant was collected for the measurement of the protein concentration using a BCA assay kit (Thermo Fisher Scientific, Waltham, MA, USA). Equal amounts of proteins (20 μg/sample) were separated via electrophoresis in sodium dodecyl sulfate–polyacrylamide gel and transferred to a PVDF membrane (Merck, Darmstadt, Germany). The membrane was blocked with Tris-buffered saline containing 0.2% (*v*/*v*) Tween-20 (TBS-T), containing 5% skim milk for 1 h at room temperature (RT), and incubated with antibodies against Tyrosinase, MITF, TRP1, TRP2 (Santa Cruz Biotechnology, Santa Cruz, CA, USA), β-actin, and GAPDH (Cell Signaling Technology, Danvers, MA, USA). After incubating the membrane with horseradish peroxidase (HRP)-conjugated secondary antibody (Thermo Fisher Scientific, Waltham, MA, USA), enhanced chemiluminescence (ECL) solution (AbClon, Seoul, Republic of Korea) was added, and signals were detected using a LuminoGraph2 (ATTO, Seoul, Republic of Korea).

### 4.4. qRT-PCR

B16F10 cells were harvested, and RNA was isolated using a Total RNA Miniprep kit (Enzynomics, Seoul, Republic of Korea). Then, cDNA was synthesized using a cDNA Synthesis kit (Enzynomics, Seoul, Republic of Korea) according to the manufacturer’s instructions. Gene expression was determined by PowerUp SYBR Green master mix (Thermo Fisher Scientific, Waltham, MA, USA) on QuantStudio 1 (Thermo Fisher Scientific, Waltham, MA, USA) according to the manufacturer’s instructions. The target primer sequences were: β-actin forward 5′-CAGCCTTCCTTCTTGGGTAT-3′, reverse 5′-TGGCATAGAGGTCTTTACGG-3′; Tyrosinase forward 5′-CTAACTTACTCAGCCCAGCATC-3′, reverse 5′-GGGTTTTGGCTTTGTCATGG-3′; MITF forward 5′-ACTTTCCCTTATCCCATCCACC-3′, reverse 5′-TGAGATCCAGAGTTGTCGTACA-3′; TRP 1 forward 5′-GATGTCTGCACTGATGACTTG-3′, reverse 5′-CCTGATTGGTCCACCCTCAG-3′; TRP 2 forward 5′-TCCTTTGCGTTGCCCTACT-3′, reverse 5′-ACCCGGCGGTTGTAGTCAT-3′. The gene expression was normalized with β-actin.

### 4.5. Melanin Measurement

B16F10 cells (1 × 10^5^) were seeded in a 24-well plate and incubated for 16 h. The cells were treated with α-MSH (Sigma-Aldrich, Darmstadt, Germany) and peptides for 72 h. The cells were incubated at 37 °C in 5% CO_2_. After 72 h of incubation, the cells were washed with phosphate-buffered saline (PBS) and lysed with 1 N NaOH containing 10% dimethyl sulfoxide (DMSO) at 60 °C for 1 h. Then, melanin levels were determined by measuring the absorbance at 405 nm using a microplate reader [48,49]. Peptides were aliquoted to different tubes and stored at −20 °C, 25 °C, and 37 °C for 45 days to test the stability of the peptide. B16F10 cells were then treated with different concentrations of peptides, and melanin contents were measured to check the stability of DualPep-Shine.

### 4.6. Luciferase Reporter Assay

B16F10 cells (1 × 10^5^) were seeded in a 24-well plate and incubated for 16 h. MITF-M (−1143 to +48) reporter construct was generated using pGL3-basic firefly luciferase reporter and co-transfected with the Renilla control vector for normalization. Then, 24 h after transfection, cells were treated with α-MSH and indicated concentrations of peptides for 8 h. Cell extracts were subjected to a dual luciferase reporter assay (Promega, Madison, WI, USA).

### 4.7. Measurement of Whitening Effect Using a 3D Human Skin Model, Neoderm-ME

Neoderm-ME (Tegoscience, Seoul, Republic of Korea), a reconstructed human 3D skin model containing melanocytes, was maintained according to the manufacturer’s instructions. Neoderm-ME was removed from the medium-containing agar, transferred onto 12-well plates, and incubated at 37 °C in 5% CO_2_ for one day. The 3D skin model was treated with each sample for 6 days. The samples and media were changed every 3 days, and the plate was incubated at 37 °C in 5% CO_2_. After 6 days, melanin content was measured at 405 nm from the tissue extract, and 3D model tissue was dissolved in 300 μL of 1 N NaOH for 45 min at 95 °C [50,51]. Fontana–Masson staining was performed after the samples were fixed in 4% paraformaldehyde overnight.

### 4.8. Penetration Assay

B16F10 cells were seeded in a 24-well plate and treated with 2.5 μM of FITC conjugated TAT, RMNE1, or DualPep-Shine for 2 h. After washing the cells with PBS 3 times, 150 μL of trypsin was used to remove membrane-bound peptides. An excess amount of serum-containing medium was added to neutralize the trypsin and was washed with FACS buffer containing 1% bovine serum albumin (BSA) and 0.1% sodium azide. Penetrated peptides were evaluated via flow cytometry by measuring FITC levels. B16F10 cells were seeded in a 24-well plate containing a glass coverslip to check the localization of the penetrated peptides. The cells were treated with FITC-conjugated peptides for 2 h and stained with Hoechst 33342 (Thermo Fisher Scientific, Waltham, MA, USA) for 5 min. The cells were washed again with PBS five times, mounted on a slide glass using gel/mount solution (Biomeda, Foster City, CA, USA), and the penetrated peptides were visualized using a fluorescence microscope [52]. To test the penetrating ability on Neoderm-ME, FITC-conjugated peptides were treated on Neoderm-ME for 24 h. After washing with PBS for three times, the tissues were fixed, frozen in OCT compound, and sectioned. The slides were washed with water before staining with Hoechst 33342 for 5 min. Sample images were taken using a confocal microscope.

### 4.9. Franz Diffusion Cells Method

The skin permeability experiments were conducted using a static type of Franz diffusion cell (B&C tech, Daejeon, Republic of Korea) according to the Franz cell method presented in OECD guideline 428. Strat-M^®^ membranes (Merck Millipore, Darmstadt, Germany)—a synthetic, non-animal-based model for transdermal diffusion testing predictive of diffusion in human skin—were placed between the donor and receptor chambers of the Franz cells. The shiny side of the membrane was facing the donor chamber, and the compartments were clamped together. The receptor chambers were filled with 8 mL of phosphate-buffered saline (Biosesang, Seongnam, Republic of Korea). The receptor fluid was stirred continuously at 600 rpm using a magnetic bar. The receptor fluid and skin temperatures were maintained at 37 ± 0.5 °C using a circulating water bath, the humidity was maintained at 30–70%, and the experiments were performed after equilibration for 30 min. TAT and peptide samples conjugated with FITC were added to the donor compartment, and light was blocked using a foil. After 24 h, 0.5 mL of receptor fluid was withdrawn through the sampling port, and the skin permeability was analyzed using a fluorescence reader [53,54].

### 4.10. Statistics

Results are presented as mean ± SD, and statistical significance was analyzed with Student’s *t*-tests. *p* < 0.05 was considered to be statistically significant for differences between the two groups.

## 5. Patent

E.C.S., C.P., Y.S. and S.C. are listed on the patent entitled “Composition comprising a peptide having skin whitening activity” (submission date: 31 August 2022; application number: 10-2022-0109704; country: the Republic of Korea).

## Data Availability

All data relevant to the study are included in the article.
